# The impact of sarcopenia on overall survival in patients with pan-RAS wild-type colorectal liver metastasis receiving hepatectomy

**DOI:** 10.1038/s41598-023-33439-x

**Published:** 2023-04-27

**Authors:** Yao-Ren Yang, Chung-Sheng Shi, Sheng-Wei Chang, Yu-Ying Wu, Yu-Li Su, Geng-Ping Lin, Feng-Che Kuan

**Affiliations:** 1 Division of Hematology and Oncology, Department of Medicine, Chang-Gung Memorial Hospital Chiayi Branch, Chiayi, Taiwan; 2 Division of Colon and Rectal Surgery, Department of Surgery, Chang Gung Memorial Hospital Chiayi Branch, Chiayi, Taiwan; 3 Department of Radiology, Chang Gung Memorial Hospital Chiayi Branch, Chiayi, Taiwan; 4 Division of Hematology Oncology, Department of Internal Medicine, Kaohsiung Chang Gung Memorial Hospital and Chang Gung University, College of Medicine, Kaohsiung, Taiwan; 5 Division of Colon and Rectal Surgery, Department of Surgery, Chang Gung Memorial Hospital, College of Medicine, Chang Gung University, Taoyuan, 33305 Taiwan

**Keywords:** Cancer, Health care, Risk factors

## Abstract

Sarcopenia has been associated with conventional chemotherapy-related toxicity, postoperative complications and poor overall survival in patients with genotype-unselected metastatic colorectal cancer (mCRC). This study aimed to evaluate the prognostic implications of sarcopenia and its change after perioperative cetuximab plus doublet chemotherapy and hepatectomy in patients with RAS wild-type colorectal liver metastasis (CRLM). Patients with CRLM from 2007 to 2018 in Chang Gung Research Database were retrospectively analyzed. Baseline characteristics as well as skeletal muscle index (SMI) at baseline and dynamic changes after interventions were collected. A multivariate Cox proportional hazard model was used to evaluate the effect of each parameter on overall survival (OS), and the Kaplan–Meier method was used to establish survival curves. A two-sided p value < 0.05 was considered statistically significance. Of 214 RAS wild-type mCRC patients who received both cetuximab and doublet chemotherapy, 77 who received upfront or subsequent hepatectomy were included in this study. The median follow-up time was 2.3 years. The rate of sarcopenia was higher in the patients who received neoadjuvant cetuximab-containing regimens than in those who received upfront hepatectomy (95% versus 63%, p = 0.001). Increased SMI after perioperative systemic therapy remained independently associated with better OS in multivariate analysis [hazard ratio (HR) = 0.27/10% increase, p = 0.013). The patients with sarcopenia had a trend of worse OS than those without sarcopenia (median OS: 4.5 versus 3.6 years, log-rank p = 0.282). Improvement in sarcopenia ([SMI after intervention − initial SMI]/initial SMI × 100%) is an important prognostic factor for OS. Future research is warranted to investigate direct interventions for sarcopenia and the impact on OS.

## Introduction

Colorectal cancer (CRC) is the third most common cancer worldwide with the second highest mortality rate^1,2^. The overall survival (OS) of metastatic colorectal cancer (mCRC) has been extended from 21 to 26–28 months after the addition of biologics to chemotherapy doublet^3–5^. The most common site of CRC metastasis is the liver, and surgical resection can further extend survival and is the only potential curative treatment for colorectal cancer with liver metastasis (CRLM)^6,7^. Cetuximab, a monoclonal antibody targeting epidermal growth factor receptor (EGFR) signaling pathway, can improve the response rate, depth of response and conversion rate in patients with oncogene in the rat sarcoma virus (RAS) wild-type mCRC^8,9^.

Sarcopenia is characterized by loss of skeletal muscle mass plus impaired muscle strength or physical performance^10^, and it has been suggested to potentially be a poor prognostic factor for OS in both non-metastatic CRC and mCRC^11–17^. Higher postoperative complications, especially infectious complications, have been shown in CRC patients with sarcopenia^16,18,19^. In addition, sarcopenic patients are susceptible to the toxicity of chemotherapy, and higher rates of grade 3 and 4 toxicity, especially neutropenia, have been reported in patients with both non-metastatic CRC and mCRC^20,21^. A recent meta-analysis of studies with mostly conventional chemotherapy in RAS-unselected patients found that sarcopenia was associated with poorer OS in patients with CRLM after local therapy^22^.

However, whether sarcopenia is a prognostic or predictive factor for RAS wild-type patients with CRLM undergoing liver resection and perioperative cetuximab-based treatment is largely unknown. Therefore, the aim of this retrospective study was to evaluate the prognostic and predictive roles of sarcopenia in mCRC and its treatment.

## Materials and methods

### Data collection

This study was approved by the Ethics Committees of Chang Gung Memorial Hospital (CGMH-IRB No.201900703B0A3). Institutional Review Broad (IRB) approval was obtained, which included a waiver of individual patient consent. And the methods in this retrospective study were carried out in accordance with the approved guideline and regulation.

Patients who were initially diagnosed with CRLM were identified from the Chang Gung Research Database (CGRD)^23^. Medical records were reviewed and patients who met the following criteria were enrolled: (1) CRLM with wild-type pan-RAS (including mutations of exon 2, 3, 4 in KRAS and NRAS), and (2) receiving cetuximab-containing regimens (FOLFOX/FOLFIRI) within 180 days after diagnosis and hepatic metastasectomy, including either upfront or subsequent hepatectomy. Patients without complete staging information, including abdominal and chest computed tomography and colonoscopy were excluded. Those without pathologic evidence of colorectal adenocarcinoma were also excluded. The included patients were arbitrarily divided into two groups: those who received neoadjuvant cetuximab plus doublet chemotherapy (CT) followed by hepatic (H) metastasectomy (CT-H group, n = 45), and those who received upfront hepatic metastasectomy followed by cetuximab plus doublet chemotherapy (H-CT group, n = 32).

The patients’ demographic data were collected including sex and age (< 60 years or ≥ 60 years). Laboratory data at the initial diagnosis, including CEA (< 20 ng/ml or ≥ 20 ng/ml) and albumin (< 3.5 g/dl or ≥ 3.5 g/dl) were also recorded. Computed tomography taken within 3 months of the initial diagnosis was used to evaluate baseline skeletal muscle area (SMA) and liver tumor burden. Corresponding follow-up images were collected at around 3 months after cetuximab plus doublet chemotherapy or hepatic metastasectomy to compare changes in muscle mass.

### Diagnosis of sarcopenia: measurement of muscle mass

Two consecutive computed tomographic cross-sectional images extending from L3 to the iliac crest were used to measure SMA. A Hounsfield unit (HU) between − 29 and + 150 was identified as skeletal muscle^24^. Cross-sectional areas of all skeletal muscles, including the psoas muscle, abdominal wall muscles (rectus abdominus, internal and external obliques and transversus abdominus), and paraspinal muscles (erector spinae and quadratus lumborum) were summed. L3 skeletal muscle index (SMI) was calculated as all SMA/height (cm^2^/m^2^), and the cut-off point for sarcopenia was defined according to Prado’s study (38.5 cm^2^/m^2^ for females and 52.4 cm^2^/m^2^ for males)^24^. The change in SMI was defined as (SMI after intervention − initial SMI)/initial SMI × 100%.

### Statistical analysis

Pearson’s chi-square test was used to compare differences between the two groups (H-CT group and CT-H group). OS was calculated from the date of initial diagnosis of CRLM to the date of death or loss of follow-up, whichever occurred first. The Kaplan–Meier method was used to establish survival curves, and the log-rank test was used to compare different curves. A multivariate Cox proportional hazard model was used to evaluate the effect of each parameter on OS. We considered a two-sided p value < 0.05 as statistically significance. All analyses were conducted using SAS version 9.4 (SAS Institute, Cary, NC, USA).

## Results

**Figure 1:**
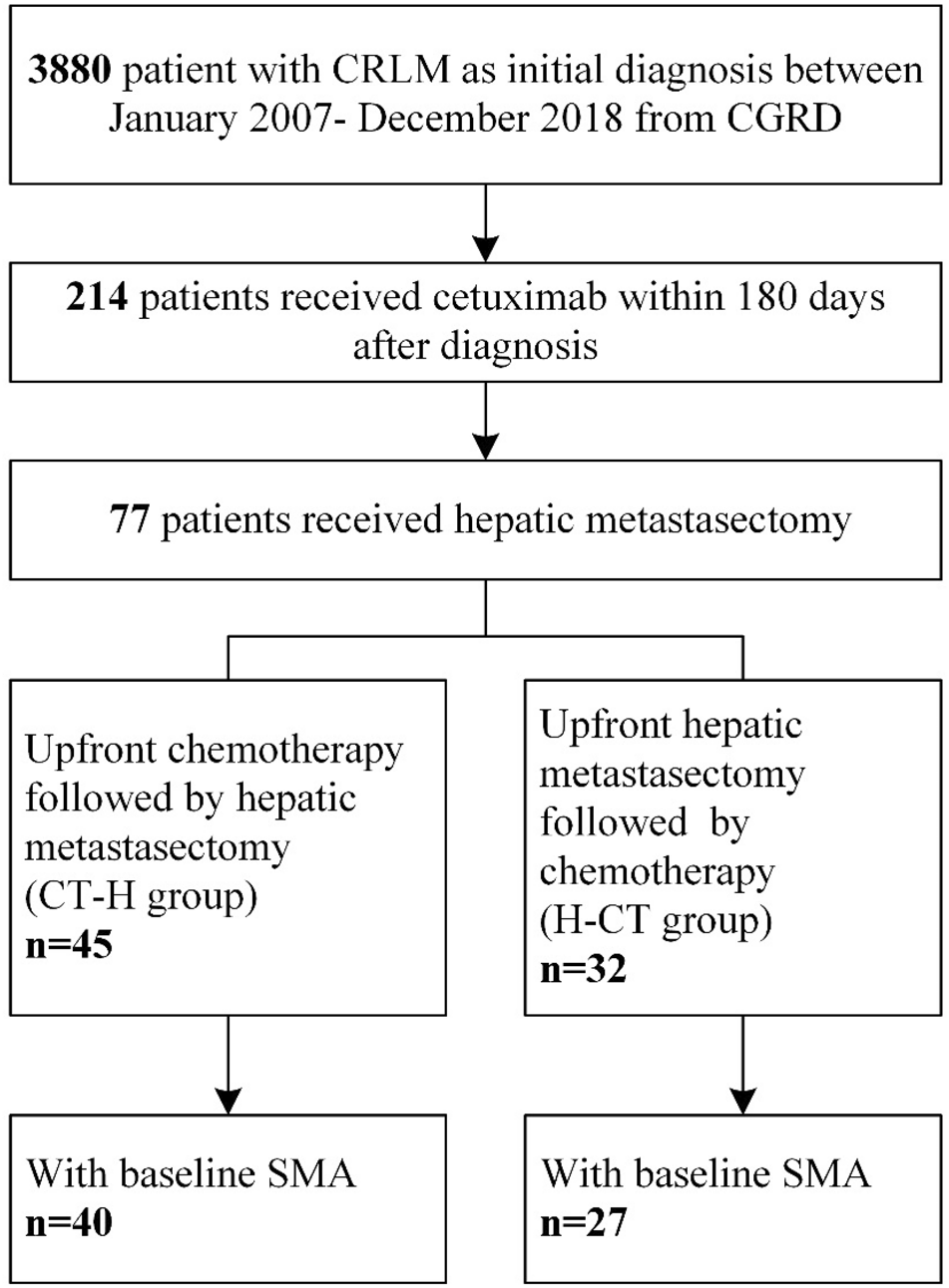
Step-wise inclusion process.

A total of 3,880 patients who were initially diagnosed with colorectal cancer and liver metastasis between January 2007 and January 2018 were identified in the CGRD (Fig. 1). Frontline cetuximab was not reimbursed by the Taiwan National Health Insurance program until December 2012. Of the 3,880 patients, 214 had wild-type RAS and received both cetuximab and doublet chemotherapy in a perioperative setting. Among these patients, 77 received hepatic metastasectomy. There were 45 and 32 patients in the CT-H and H-CT groups, respectively, of whom 40 and 27 had baseline and follow-up images.

### Baseline characteristics

The demographics of the included patients are shown in Table 1. The baseline characteristics of the CT-H and H-CT groups were similar in sex, age, tumor sidedness, serum albumin and serum CEA, except for sarcopenia. Patients in the CT-H group had a significantly higher rate of sarcopenia (CT-H versus H-CT group: 95% versus 63%, p = 0.001). In addition, higher volume percentage of liver occupied by metastatic lesions have relatively lower initial SMI (Supplement Figs. 1 and 2).

**Table 1:** Clinical characteristics of CT-H and H-CT patients.

Variables	All		CT-H		H-CT		p value
	n	%	n	%	n	%	
Total	77		45		32		
Sex							0.389
Female	27	35.1	14	31.1	13	40.6	
Male	50	64.9	31	68.9	19	59.4	
Age, years							0.082
< 60	45	58.4	30	66.7	15	46.9	
≥ 60	32	41.6	15	33.7	17	53.1	
Sidedness of colon							0.422
Left	46	59.7	25	55.6	21	65.6	
Right	8	10.4	4	8.9	7	21.9	
Rectal	23	29.9	16	35.6	4	12.5	
Albumin, g/dl							0.855
< 3.5	43	61.4	28	62.2	15	60.0	
≥ 3.5	27	38.6	17	37.8	10	40.0	
CEA, ng/ml							0.887
< 20	49	73.1	29	72.5	20	74.1	
≥ 20	18	26.9	11	27.5	7	25.9	
Sarcopenia							0.001
No	12	17.9	2	5.0	10	37.0	
Yes	55	82.1	38	95.0	17	63.0	

	Mean	SD	Mean	SD	Mean	SD	
SMI change after CT, %	− 1.3	16.0	− 1.7	17.3	− 0.7	14.3	0.803
SMI change after OP, %	− 5.4	17.2	− 3.5	17.7	− 14.8	11.4	0.094

The mean changes in SMI in the CT-H and H-CT groups after cetuximab plus doublet chemotherapy were − 1.7% and − 0.7%, respectively (p = 0.803), and the mean changes after hepatectomy were − 3.5% and − 14.8%, respectively (p = 0.094).

### Prognostic factors for overall survival

**Figure 2:**
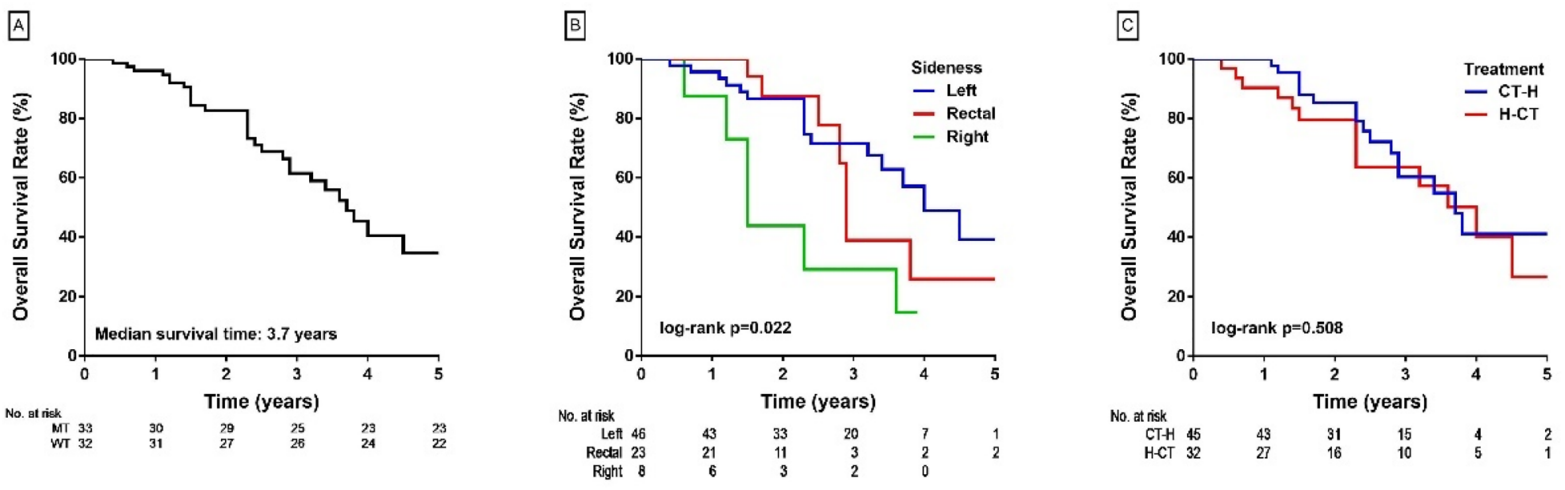
(**A**) Median survival time for all patients: 3.7 years. (**B**) Median survival time for the patients with left-sided tumors: 4 years; rectal tumors: 2.9 years; right-sided tumors: 1.5 years; (**C**) median survival time for the CT-H group: 3.7 years and H-CT group: 4.0 years.

The median OS of the patients was 3.7 years, with a median follow-up of 2.3 years (Fig. 2A). In univariate analysis of OS, tumor sidedness [left-sided and rectum versus right-sided cancer] (p = 0.009 and p = 0.048, respectively), and the changes in SMI after chemotherapy (HR: 0.72 per 10-unit increase in SMI, p = 0.027) and hepatectomy (HR: 0.72 per 10-unit increase of SMI, p = 0.028) were significant prognostic factors. In multivariate Cox proportional hazard analysis, improvement in SMI after chemotherapy remained the only significant prognostic factor for OS (HR: 0.27, p = 0.013) (Table 2).

**Table 2:** Univariate and multivariate analysis of prognostic factors for overall survival.

Variables	Crude HR		p value	adjusted HR		p value
	HR	95% CI		HR	95% CI	
Gender						
Female	Ref			Ref		
Male	1.25	(0.58–2.69)	0.574	1.18	(0.18–7.88)	0.866
Age, years						
< 60	Ref			Ref		
≥ 60	0.66	(0.31–1.41)	0.282	0.64	(0.11–3.70)	0.622
Sidedness of tumor						
Left-sided colon	0.28	(0.11–0.73)	0.009	0.06	(0.01–1.06)	0.054
Right-sided colon	Ref			Ref		
Rectal	0.33	(0.11–0.99)	0.048	0.70	(0.05–9.46)	0.789
Albumin, g/dl						
< 3.5	Ref			Ref		
≥ 3.5	1.00	(0.47–2.14)	0.998	0.38	(0.07–2.06)	0.263
CEA, ng/ml						
< 20	Ref			Ref		
≥ 20	0.98	(0.41–2.38)	0.967	1.58	(0.21–11.8)	0.654
Sarcopenia						
No	Ref			Ref		
Yes	1.92	(0.57–6.44)	0.293	3.48	(0.26–47.2)	0.349
Treatment						
CT-H	0.78	(0.37–1.62)	0.505	0.35	(0.05–2.32)	0.274
H-CT	Ref			Ref		
SMI change after CT*	0.72	(0.53–0.96)	0.027	0.27	(0.10–0.76)	0.013
SMI change after OP*	0.72	(0.54–0.97)	0.028	1.52	(0.77–2.97)	0.226

*HRs were calculated by per 10% increase of SMI.

**Figure 3:**
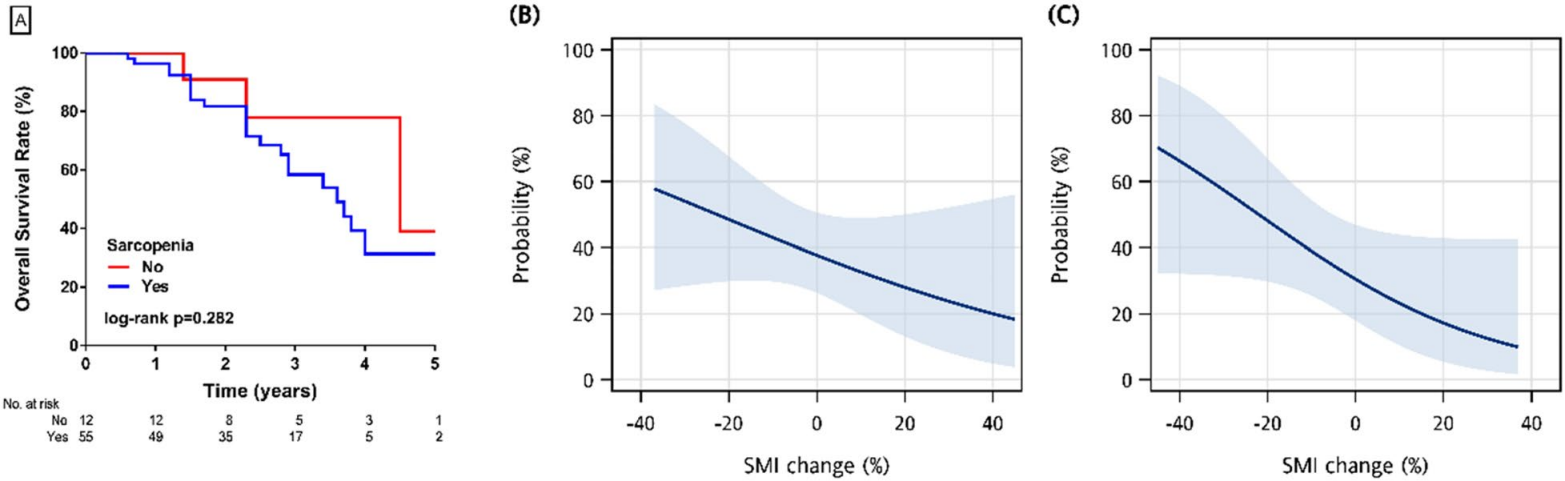
(**A**) Median survival time of the patients with sarcopenia: 3.6 years: without sarcopenia: 4.5 years; (**B**) probability of death by percentage change in SMI after chemotherapy; (**C**) probability of death by percentage change in SMI after hepatectomy.

The OS rates of the patients with left-sided colon and rectal cancers were better than that of the patients with right-sided cancers (median OS: 4 and 2.9 years versus 1.5 years, log-rank p = 0.022) (Fig. 2B). There was no significant difference in OS between the CT-H and H-CT groups (log-rank p = 0.508) (Fig. 2C). There was a trend of better OS in the patients without sarcopenia (4.5 versus 3.6 years, log-rank p = 0.282) (Fig. 3A). The magnitude of change in SMI was positively correlated with OS (Fig. 3B,C).

## Discussion

To the best of our knowledge, this is the first study to show a positive correlation between increased OS and SMI after perioperative treatment with a cetuximab-containing regimen in patients with RAS wild-type CRLM. A previous study reported an independent association between losing more than 9% of skeletal muscle per 3 months during palliative chemotherapy with poor OS. However, none of the enrolled patients received a cetuximab-containing regimen, and RAS mutation status was not reported^15^. While both cetuximab combined with doublet chemotherapy and hepatic metastasectomy can improve the outcomes of mCRC, we focused on the prognostic implications of sarcopenia and its change after treatment in this study. Our results showed that change in sarcopenia was a strong prognostic factor for OS in patients with RAS wild-type CRLM.

The operational definitions of sarcopenia are inconsistent across international working groups, but they are characterized by loss of muscle mass, muscle strength and performance^25–29^. Measuring the total muscle area around the third lumber vertebrae (L3) using computed tomography can strongly predict the ratio of whole-body fat to fat-free mass, and it has become a very convenient tool to evaluate secondary sarcopenia in patients with colorectal cancer^30^. The widely used cut-off point of skeletal mass loss in sarcopenia is according to Prado’s study (38.5 cm^2^/m^2^ for females and 52.4 cm^2^/m^2^ for males)^24^. The prevalence of sarcopenia in a recent meta-analysis of trials with heterogenous definitions of sarcopenia varied greatly in patients with CRLM ranging from 17 to 65%^22^, due in part to different cut-off points. In the present study, approximately 82% of the patients had sarcopenia, and surprisingly the prevalence of sarcopenia was higher in the CT-H group than in the H-CT group (95% versus 63%, p = 0.001). This could be partially because those who received upfront cetuximab-containing systemic treatment had more severe initial involvement of the liver, which could cause physiologic derangements such as anorexia, organ dysfunction and decreased activity^10^. In addition, previous studies have suggested that CRLM is associated with an altered microenvironment with high levels of inflammatory cytokines such as TNF-alpha and IL-6, which could subsequently promote sarcopenia^31,32^.

Previous studies have suggested that sarcopenia is a poor prognostic factor for OS in both non-metastatic CRC and mCRC^11–14^. However, the correlation between sarcopenia and OS is inconclusive in patients undergoing liver resection for CRLM. A recent meta-analysis concluded that sarcopenia is associated with a poorer OS based on trials including mostly RAS-unselected patients receiving conventional chemotherapy without the current widely used biologics^22^. Thus, whether this conclusion can be applied to patients with wild-type RAS receiving cetuximab is unknown. In the present study, the patients with sarcopenia had a trend of poorer OS compared to those without sarcopenia (median OS: 3.6 years versus 4.5 years, log-rank p = 0.282), and the difference (> 10 months) is of clinical significance. In addition, all eight patients with right-sided tumors had sarcopenia, which may have diluted the effect in multivariate analysis since sidedness has been reported to be an important prognostic factor in these patients^33^.

## Limitations

This study has several limitations. First, this was a retrospective study with relatively small number of patients. However, we enrolled patients receiving both perioperative cetuximab-based doublet chemotherapy and hepatectomy, which are considered to be important prognostic factors for OS in modern real-world practice. Second, sarcopenia was evaluated only by skeletal muscle mass without performance status or strength. We used the widely accepted criteria for sarcopenia, and showed a higher prevalence in patients with more severe involvement of liver metastasis. Third, there was an association between an improvement in sarcopenia and better OS, however it is not clear whether interventions for sarcopenia can impact survival itself.

## Conclusion

Improvement in sarcopenia after perioperative treatment with a cetuximab-containing regimen is an important prognostic factor for OS in patients with RAS wild-type CRLM after hepatectomy. Future research is warranted to investigate direct interventions for sarcopenia and the impact on OS.

## Supplementary Information

Supplementary Information.

## Data Availability

Data and codes can be provided by the corresponding author upon reasonable request.
