# Supplementary material for: The impact of sarcopenia on overall survival in patients with pan-RAS wild-type colorectal liver metastasis receiving hepatectomy
Source: Sci Rep. 2023 Apr 27;13:6911. doi: 10.1038/s41598-023-33439-x (PMC10140270; doi:10.1038/s41598-023-33439-x)
Supplement: Supplementary file 1 — Supplementary Information. [file 41598_2023_33439_MOESM1_ESM.docx]

**Supplement Figure 1. Liver tumor burden and skeletal muscle area**

A and B: non-sarcopenic patient with low liver tumor burden (enclosed area by green line) and high skeletal muscle area (enclosed areas by yellow line)

C and D: Sarcopenic patient with high liver tumor burden (enclosed areas by green line) and low skeletal muscle area (enclosed areas by yellow line)


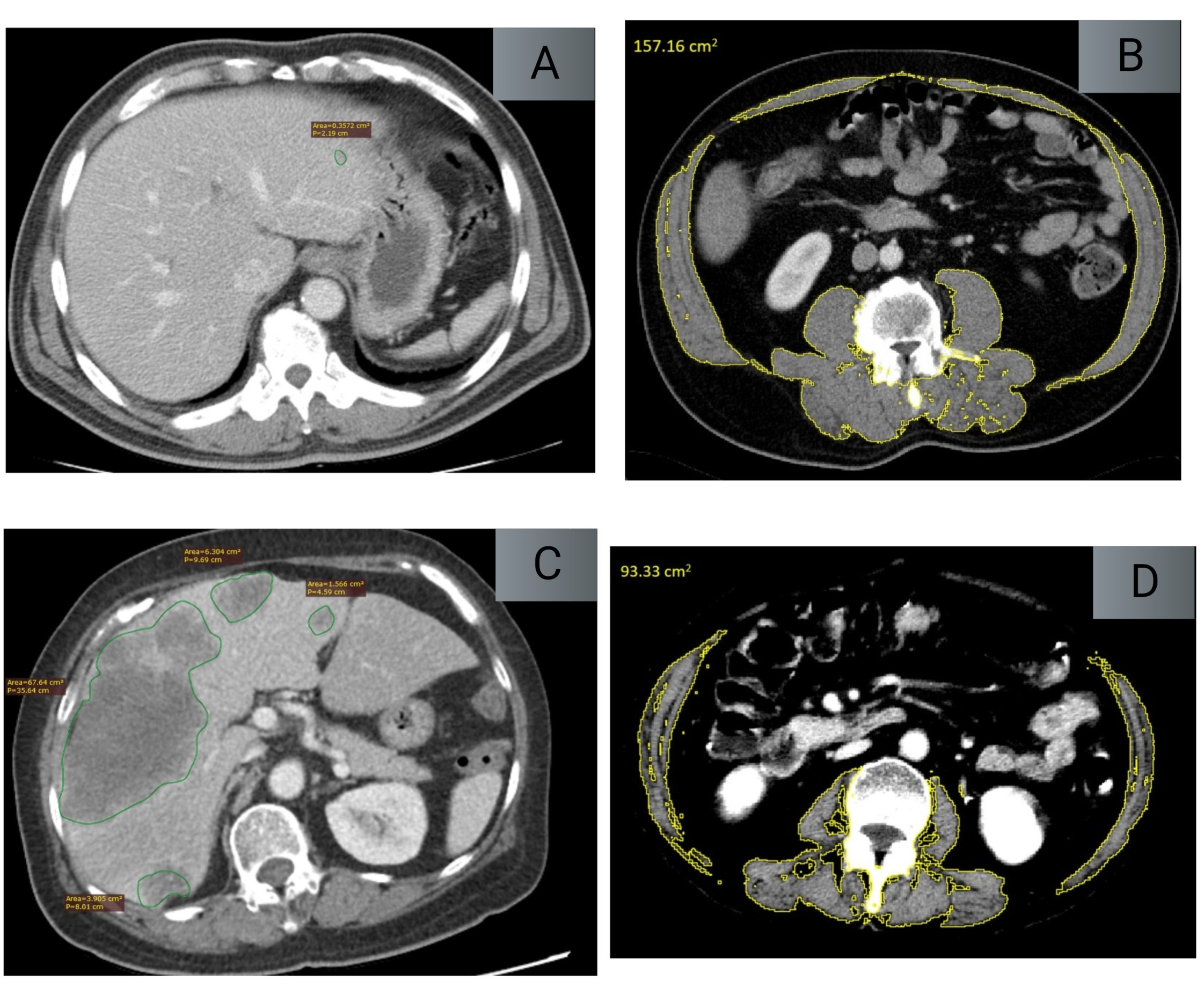


**Supplement Figure 2.**

Scatter plot of Correlation between volume percentage of liver occupied by metastasis lesion and initial SMI, grouped by two treatment sequences.

Figure shows the correlation in volume percentage of liver occupied by metastasis lesion and initial SMI. In both treatment groups, the higher volume percentage of liver occupied by metastatic lesions have lower initial SMI. Compared with H-CT group (yellow dots), there is a lower initial SMI in CT-H group (blue dots).


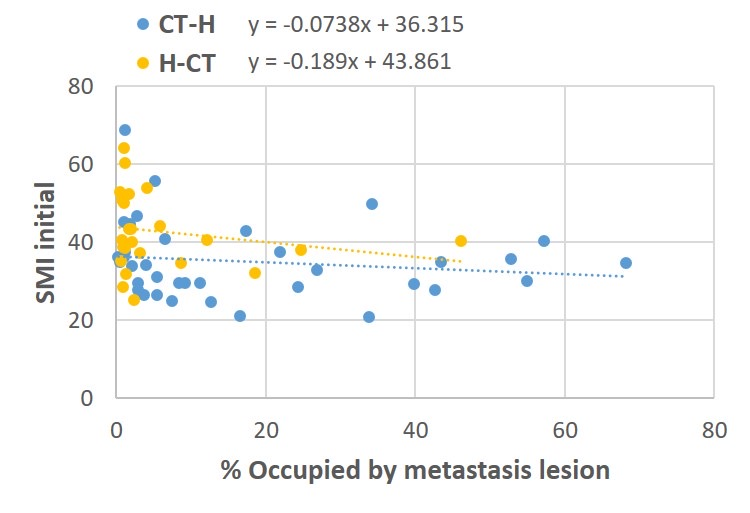


**Supplement table 1**. Liver tumor burden (liver occupation rate)

|  | N | Mean (% liver occupation) | Standard deviation |
| --- | --- | --- | --- |
| CT-H Patient | 37 | 17.0% | 19.4% |
| H-CT Patient | 25 | 5.7% | 10.32% |

**Supplement table 2**. Initial skeletal muscle index

|  | N | Mean (cm2/m2) | Standard deviation |
| --- | --- | --- | --- |
| CT-H Patient | 37 | 35 | 9.66 |
| H-CT Patient | 25 | 42 | 9.68 |
